# A Multi-Object Tracking Method for Pigs Based on Oriented Bounding Box Detection

**DOI:** 10.3390/ani16213490

**Published:** 2026-11-04

**Authors:** Xuhui Ye, Lilu Huang, Yuyan Li, Zhong Li, Yan Chen

**Affiliations:** 1 School of Mechanical Engineering, Hubei University of Technology, Wuhan 430068, China; yxh89@hbut.edu.cn (X.Y.); 102510152@hbut.edu.cn (L.H.); 2410111103@hbut.edu.cn (Y.L.); 102610148@hbut.edu.cn (Z.L.); 2 School of Mechanical and Electrical Engineering, Wuhan Donghu University, Wuhan 430212, China

**Keywords:** multi-object tracking, oriented bounding box, YOLO11m-OBB, pigs, AdaptiveSORT, knowledge distillation

## Abstract

To address background redundancy in detection boxes and identity switches caused by variable body orientations, mutual occlusion, and high appearance similarity among group-housed pigs, a multi-object tracking method based on oriented bounding box detection was proposed to improve the accuracy of pig detection and trajectory association in farming environments. First, YOLO11m-OBB was adopted as the baseline detector. Its original backbone was replaced with EfficientViT-M0, and a dual-teacher knowledge distillation framework based on the DINOv3 vision foundation model was constructed, resulting in a lightweight pig detection model named PIG-YOLO. Second, an AdaptiveSORT tracking algorithm was developed within the tracking-by-detection framework. Camera motion compensation, adaptive state prediction, and multi-scale feature extraction modules were incorporated into the multi-object tracking process, together with a three-stage association strategy and a dedicated track management mechanism. Finally, detection, tracking, and cross-dataset generalization experiments were conducted using both public and self-collected pig video datasets. PIG-YOLO achieved a precision of 94.7%, a recall of 88.3%, an mAP50 of 93.1%, and an mAP50–95 of 85.6%, representing improvements of 3.7, 9.9, 8.0, and 8.9 percentage points over the baseline model, respectively. Meanwhile, the number of parameters, floating-point operations, and model size were reduced by 37.7%, 51.5%, and 36.2%, respectively. On the public test dataset, the combination of PIG-YOLO and AdaptiveSORT achieved HOTA, MOTA, and IDF1 scores of 96.32%, 98.82%, and 99.41%, respectively. On the private dataset, the corresponding scores were 94.98%, 97.31%, and 98.86%. In the cross-dataset generalization experiment, the proposed method achieved HOTA, MOTA, and IDF1 scores of 71.231%, 76.043%, and 83.008%, respectively, outperforming the other comparative tracking algorithms in overall performance. The proposed method reduces the computational complexity of the detection model while improving the accuracy of pig detection and the stability of trajectory association. It therefore provides technical support for pig behavior analysis and intelligent monitoring in large-scale pig farms.

## Simple Summary

Monitoring individual pigs can help farmers understand their activity and identify changes that may indicate health or welfare problems. However, continuous manual observation is time-consuming, and automatic video monitoring is challenging because pigs look similar, frequently overlap, and move in different directions. This study aimed to develop a more accurate and computationally efficient method for locating pigs and following each animal throughout a video. The method uses rectangular outlines that rotate to fit each pig’s body and combines information about appearance and movement to maintain individual identities. Tests using publicly available and newly recorded farm videos showed improved detection and tracking compared with the methods evaluated. The system made no identity mix-ups in the public test videos and only one in the privately collected test videos. The detection model also required approximately half the computation of the original model. Further testing on videos from another source demonstrated its potential to work across different recording conditions. These findings provide a foundation for automated monitoring of individual pig activity, which could reduce observation workloads, support earlier recognition of unusual behavior, and help farmers make informed decisions about animal care.

## 1. Introduction

Under intensive farming conditions, the activity patterns and behavioral changes of individual pigs are closely associated with their health status. Conventional manual observation is not only time-consuming and labor-intensive but also unsuitable for the long-term, continuous, and individualized monitoring of group-housed pigs. Computer vision-based multi-object tracking can assign a persistent identity to each pig in a non-contact manner and continuously obtain information regarding its behavioral state, movement trajectory, and activity area. Consequently, this technology has increasingly become an important tool for behavioral analysis and health monitoring in group-housed pigs [1,2,3,4].

Compared with pedestrian and vehicle tracking, multi-object tracking of pigs presents more pronounced domain-specific challenges. On the one hand, pigs housed in the same pen are highly similar in body shape, coat color, and texture, limiting the discriminative value of appearance features. On the other hand, pigs frequently exhibit aggregation, physical contact, crossing movements, and prolonged occlusion, which can result in overlapping detection boxes, interrupted trajectories, and identity switches. In addition, variations in illumination between daytime and nighttime, interference from pen backgrounds, and differences in camera viewpoints can reduce model stability across farming environments. Guo et al. [5] compared several deep learning-based tracking methods and found that an appropriate integration of appearance information and spatial association information could reduce identity switches among pigs. Nevertheless, the overall performance of animal tracking remained lower than that of conventional pedestrian tracking. Zhang et al. [6] used convolutional neural networks and Kalman filtering to obtain pig locations, orientations, and movement trajectories, thereby enabling the automatic quantification of social contact behavior. However, association methods based on simple motion models remain limited under complex occlusion conditions.

To improve trajectory association under occlusion, recent studies have gradually shifted from motion-only matching toward the joint modeling of detection, motion, and appearance information. Tu et al. [7] proposed a pig behavior tracking method based on an improved DeepSORT algorithm. By optimizing the matching strategy and introducing a behavior classification module, the method achieved stable tracking of group-housed pigs under occlusion and illumination changes. Gan et al. [8] developed an online piglet tracking model based on a convolutional neural network and used the Hungarian algorithm together with distance constraints to correct erroneous state predictions. Although the model achieved high IDF1 and MOTA scores, it was not specifically designed for lightweight deployment and therefore imposed relatively high computational requirements. Huang et al. [9] proposed MPC-YD, a pig counting and tracking algorithm combining YOLOv5 with DeepSORT. By introducing a spatial pyramid pooling network and a spatial-state correction mechanism, the method improved tracking robustness during rapid movement and achieved a MOTA of 85.32% and a MOTP of 84.13%. This approach provided a useful reference for deployment on edge devices. Yu et al. [10] proposed the FTO-SORT tracking optimizer, which introduced a foreground attribute module into the BoT-SORT framework for multi-object pig tracking in previously unseen housing environments. The method achieved an IDF1 score of 90.2%, representing an improvement of 15.1 percentage points over the baseline algorithm. The publicly released dataset also supports subsequent academic research. Li et al. [11] improved a lightweight detector using an efficient attention module and combined it with ByteTrack to construct a pig behavior tracking method, thereby reducing computational complexity while improving detection and identity association performance. Collectively, these studies demonstrate that both detection completeness and the design of the association strategy determine the stability of long-term pig tracking.

Most existing methods use horizontal bounding boxes to represent pig locations. However, pigs in overhead-view farming environments are usually distributed at arbitrary orientations. Horizontal bounding boxes therefore tend to include excessive information from the floor and adjacent animals, which is unfavorable for accurately distinguishing individual pigs. Ho et al. [12] used Refined Rotation RetinaNet (R^3^Det) to localize neonatal piglets and combined it with SORT for individual tracking and movement quantification. Their method achieved MOTA, MOTP, and IDF1 scores of 97.35%, 96.97%, and 98.30%, respectively. Lu et al. [13] proposed ORP-Byte, an improved ByteTrack-based multi-object tracking algorithm combined with an Oriented RepPoints detector. The method achieved a MOTA score of 99.8% and an IDF1 score of 91.6%, with only 16 identity switches. Its rotated bounding-box mechanism substantially reduced background interference and was suitable for low-frame-rate video scenarios. Liu et al. [14] proposed a rotated bounding-box detection method for estimating pig locations and orientations. The geometric characteristics of each pig were represented using its center point, long side, short side, and orientation vector. FastSeq-NMS was then employed to integrate orientation and temporal information from adjacent frames, resulting in an AP50 of 96.54% on the test dataset. Wang et al. [15] further adopted OBB-based annotations and incorporated the consistency of pig postures before and after occlusion into BoT-SORT. ProbIoU was used to construct a matching strategy suitable for rotated bounding boxes. Their method achieved HOTA, MOTA, and IDF1 scores of 82.4%, 97.7%, and 89.1%, respectively. Lin et al. [16] proposed PigTracker, which combined sequential-vertex rotated bounding boxes with an improved Kalman filter containing an orientation state. A temporal window was also introduced to correct anomalous orientation estimates in individual frames. PigTracker achieved a HOTA score of 93.44%, which was 3.65 percentage points higher than that of HBB combined with ByteTrack. Hu et al. [17] proposed a lightweight method for detecting pig posture using oriented bounding boxes (OBBs), targeting deployment on edge devices. Based on YOLO11m-OBB, the method replaced the original backbone with MobileNetV3-Large and incorporated shuffle attention and a bidirectional feature pyramid to balance model compactness and feature extraction capability. However, it addressed only single-frame detection and did not continuously track individual pigs, indicating a need for further research into multi-object tracking in group-housing environments. Collectively, these studies indicate that the completeness of detection results and the effectiveness of association strategies jointly determine the stability of long-term pig tracking.

Despite these advances, several limitations remain. First, pig detection models generally require a trade-off between detection accuracy and model size, while lightweight design may lead to the loss of occluded targets and small-scale behavioral features. Second, existing rotated-object tracking methods primarily focus on geometric matching between bounding boxes and make limited use of the general semantic knowledge contained in vision foundation models. Third, under prolonged occlusion, abrupt changes in motion state, and camera-view variations, reliance on a single-stage motion- or appearance-based association strategy can still result in incorrect matches. Fourth, some studies have been evaluated only in a single environment, without systematic assessment using public datasets, private datasets, and cross-domain conditions.

## 2. Data Acquisition and Processing

### 2.1. Data Acquisition

Both public and private datasets were used in this study. The public dataset, provided by Posta et al. [18], contains ten video sequences, of which five were used for training and validation and the remaining five were used for testing. The private dataset was collected at a commercial pig farm in Hebi City, China, and contains thirty 1-min video sequences, of which twenty-five were used for training and validation and five were used for testing. All videos were captured using fixed overhead cameras (DS-2DC3A40IW-DE; Hangzhou Hikvision Digital Technology Co., Ltd., Hangzhou, China) at a resolution of 2688 × 1520 pixels. The videos were sampled and annotated at 5 frames/s, allowing the activity and behavior of pigs within the pens to be recorded consistently.

To improve the applicability of the pig behavior analysis model in actual farming environments, the datasets covered a wide range of pig breeds, age groups, body-weight ranges, stocking densities, and housing conditions. The datasets included Large White pigs, Xiao’er Hua pigs, and crossbred Xiao’er Hua × Tibetan pigs. According to the growth stage, the pigs were divided into nursery, growing, and finishing groups, with age ranges of 4–11, 12–19, and 20–27 weeks, respectively. The corresponding body-weight ranges were approximately (7, 25] kg, (25, 70] kg, and (70, 130] kg. Based on manual observations, pig activity intensity was further classified into high and low levels to reflect differences in movement among physiological stages and environmental conditions. The datasets also covered several typical flooring conditions, including concrete floors, metal slatted floors, and plastic slatted floors. Because these floor types differ in texture, reflectivity, and background-edge characteristics, their inclusion facilitates evaluation of the model’s adaptability to complex backgrounds. The temperature and humidity of the data acquisition environments were maintained within appropriate ranges, allowing the pigs to behave naturally and thereby ensuring the authenticity and validity of the behavioral samples. In addition to conventional daytime conditions, the datasets included strong illumination, normal illumination, nighttime imaging, densely distributed targets, and sparsely distributed targets. These conditions were used to evaluate the detection and identification performance of the algorithm under illumination disturbances, occlusion, and variations in animal density. Detailed information on the test datasets is presented in Table 1.

To standardize the classification criteria for the scene characteristics listed in Table 1, daytime is defined as 06:00–18:00 local time at the recording site, and all other times are classified as nighttime. Group density is quantified using the video-level mean target occupancy ratio, defined as the frame-wise average of the fraction of the effective pen area covered by the union of the pigs’ oriented bounding boxes. Scenes with a mean occupancy ratio below 30% are classified as sparse; otherwise, they are classified as dense. Activity level is determined from manually annotated trajectories. For each individual, normalized movement speed is calculated by dividing its center-point movement speed by the mean long-side length of its oriented bounding box. This normalized speed is then averaged across individuals and valid frames within each video. Activity is classified as low when the mean normalized speed is below 0.10 body lengths/s and high otherwise.

### 2.2. Data Processing

**Figure 1:**
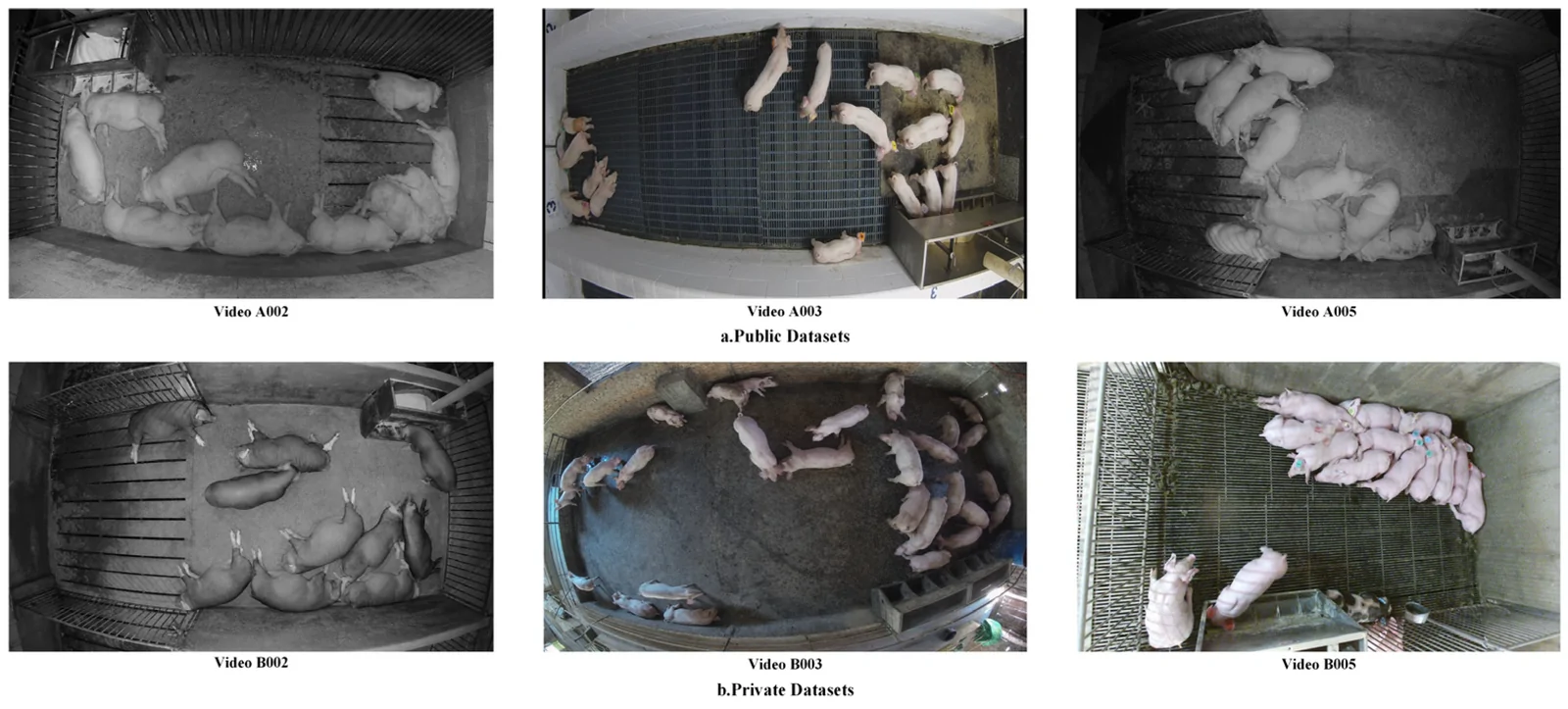
Representative images of pigs in the datasets.

Common annotation formats for object detection include horizontal bounding boxes (HBBs) [19] and oriented bounding boxes (OBBs) [20]. HBBs use axis-aligned rectangles and are suitable for regularly shaped objects. However, when pigs are obliquely oriented, touching one another, or irregular in outline, HBBs tend to include substantial background information, which can reduce detection accuracy. OBBs can be rotated to align with object orientations, fitting the contours of pig bodies more closely and thereby reducing background interference and improving detection and tracking performance. Accordingly, keyframes were extracted from the videos using FFmpeg 6.0, and 2442 images were selected for annotation. Individual pigs were annotated using OBBs in Labelme(version 7.9.1; https://github.com/wkentaro/labelme, accessed on 25 October 2026), and their object IDs and bounding-box coordinates were recorded. Representative examples from the dataset are shown in Figure 1.

To ensure the accuracy of pig behavior classification, the definitions of pig posture and feeding behavior were established with reference to previous studies on group-housed pigs [21,22,23] and were supplemented by manual observations. The annotated behavioral categories included standing, lateral lying, and feeding. The specific behavioral categories and their corresponding criteria are presented in Table 2. Furthermore, to construct sequence-level ground-truth annotations for the multi-object tracking experiments, the Computer Vision Annotation Tool (CVAT; version 2.81.0; https://github.com/cvat-ai/cvat, accessed on 25 October 2026) was used to annotate the pig video sequences in MOT16 format. The resulting multi-object tracking annotation files contained the frame index, target bounding-box coordinates, and track ID, providing the ground-truth data required for evaluating the performance of the subsequent tracking algorithm. The annotated images were divided into training, validation, and test sets at a ratio of 8:1:1, comprising 1954, 244, and 244 images, respectively, as detailed in Table 3.

## 3. Pig Multi-Object Tracking Method

**Figure 2:**
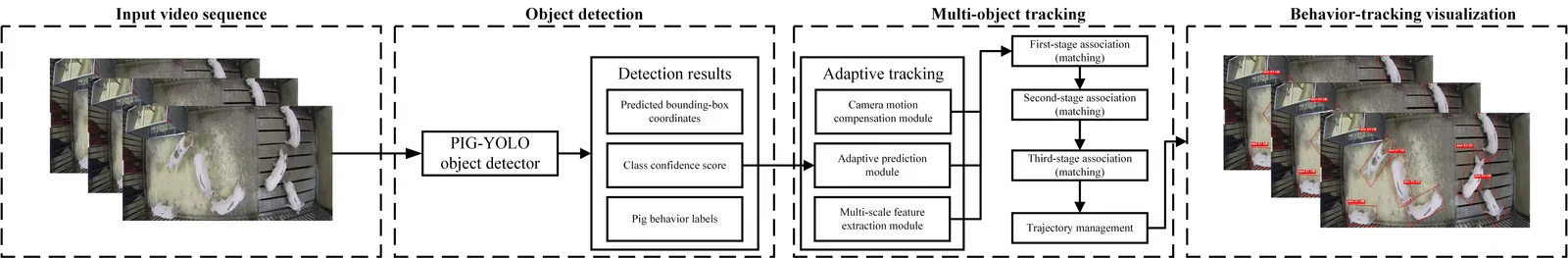
Workflow of the proposed pig multi-object tracking method.

The workflow of the proposed pig multi-object tracking method is illustrated in Figure 2, with the schematic design informed by previous research [17]. First, the developed PIG-YOLO detector is used to detect pigs in the input image sequence. The resulting detections are then passed to the AdaptiveSORT tracking algorithm developed in this study. After three stages of data association, an additional matching operation is performed during track management, followed by track initialization, updating, and termination. Finally, the generated trajectories and corresponding object identities are used to produce the tracking results.

### 3.1. Improved Pig Detection Model

**Figure 3:**
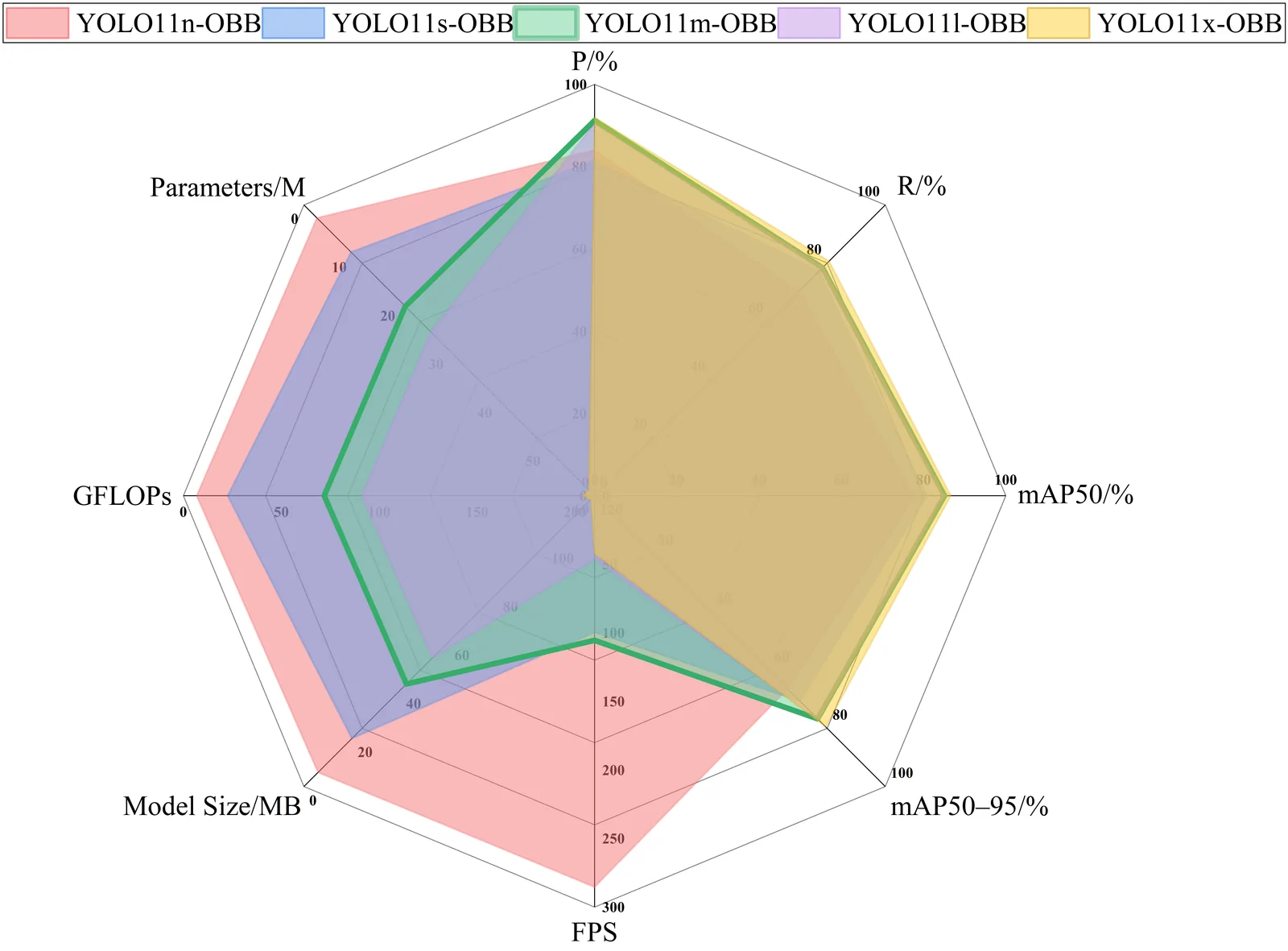
Test results of the five YOLO11-OBB variants.

YOLO11 was released by the Ultralytics team in September 2024 [24]. Compared with YOLOv8, YOLO11 introduces the C3k2 and C2PSA modules and employs lightweight depthwise-separable convolutions in the classification branch of its anchor-free decoupled detection head. These architectural modifications improve feature representation and parameter efficiency, enabling YOLO11 to achieve a favorable balance between detection accuracy and computational efficiency. Oriented bounding box detection can more accurately fit pig body regions at arbitrary orientations and reduce interference from irrelevant background pixels, thereby improving detection accuracy. YOLO11-OBB is the oriented-object detection variant of YOLO11 and is available in five model scales: n, s, m, l, and x. The performance of these five variants on the pig dataset is presented in Figure 3.

As shown in Figure 3, YOLO11m-OBB provides the most favorable trade-off between detection accuracy and model complexity among the five variants. Therefore, YOLO11m-OBB was selected as the baseline model. To further reduce computational complexity while enhancing the representation of pig-specific features, two major improvements were introduced into YOLO11m-OBB. First, the original backbone was replaced with the lightweight EfficientViT-M0 network. Its efficient feature extraction operations and cascaded group attention mechanism enhance the extraction of both local details and global semantic information while reducing the number of model parameters and computational operations. Second, a dual-teacher knowledge distillation framework based on the DINOv3 vision foundation model was constructed. YOLO11x-OBB was employed as the task teacher to transfer task-specific knowledge related to classification and oriented bounding box regression. Meanwhile, DINOv3 ViT-S/16 was used as the feature teacher to provide dense semantic supervision at multiple feature levels. The complementary supervision from the two teacher models improves the detection accuracy and generalization capability of the lightweight student model.

**Figure 4:**
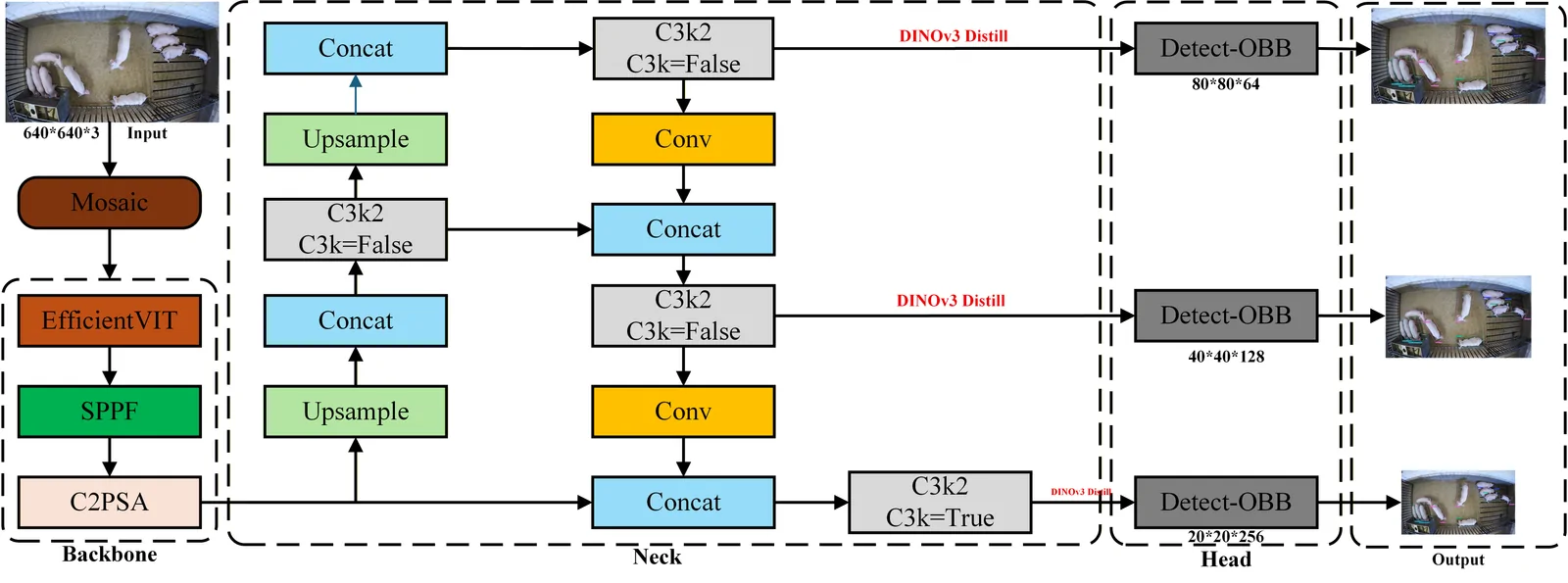
Overall architecture of the PIG-YOLO model.

The resulting improved detection model is referred to as PIG-YOLO, and its overall architecture is illustrated in Figure 4.

#### 3.1.1. Efficientvit Lightweight Backbone Network

**Figure 5:**
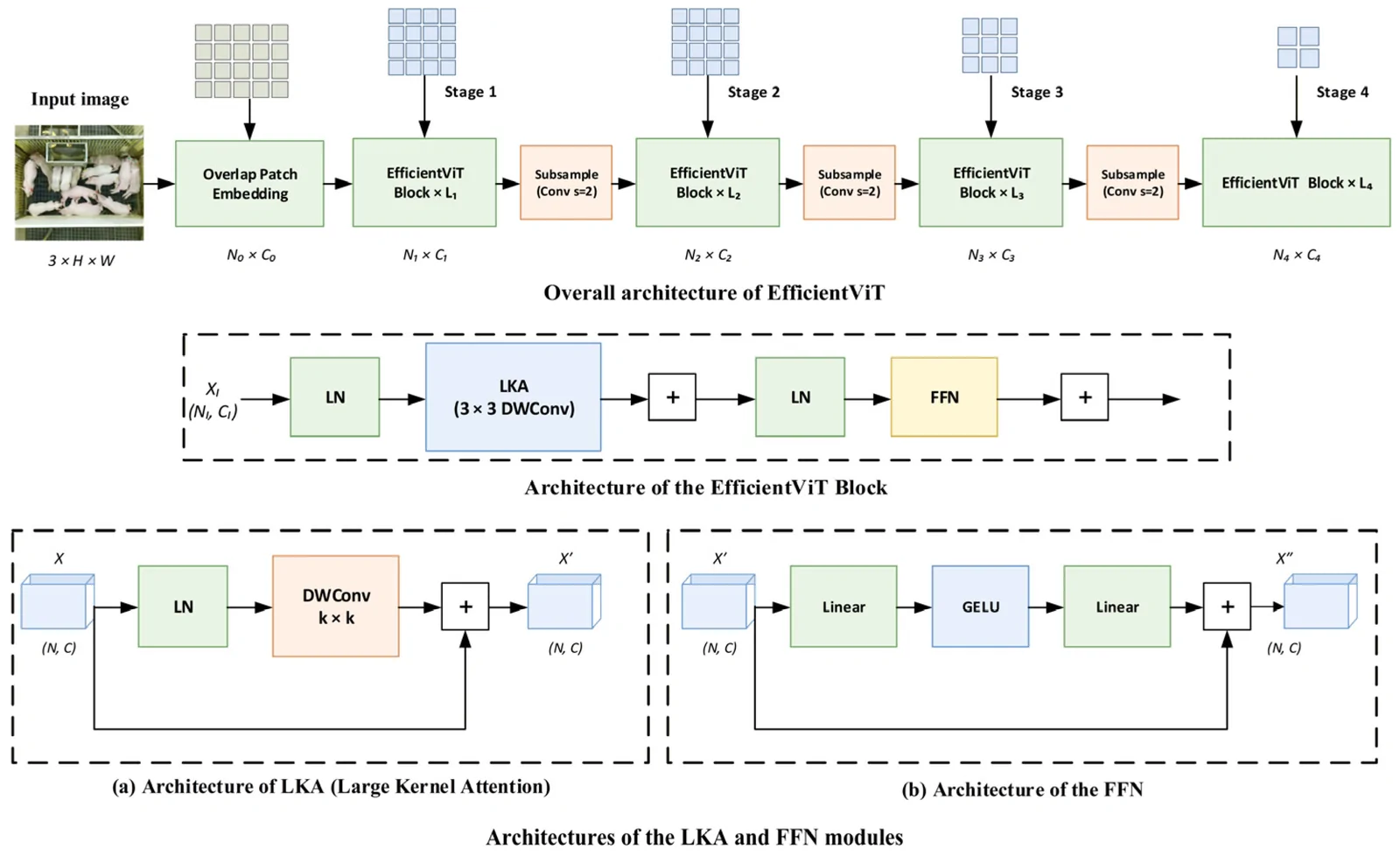
Architecture of EfficientViT.

EfficientViT is a lightweight vision Transformer designed for efficient visual processing [25], and its overall architecture is illustrated in Figure 5. To address the high computational complexity and memory-access overhead of conventional vision Transformers, EfficientViT adopts a sandwich architecture in which a single self-attention layer is embedded between two feed-forward networks. This design reduces the computational cost of attention while enhancing information interaction across channels. Its cascaded group attention mechanism divides the input features into multiple subspaces and progressively propagates the features generated by different attention heads in a cascaded manner, thereby reducing redundant computation in multi-head attention.

In this study, EfficientViT-M0 was used to replace the original backbone network of YOLO11m-OBB. Convolutional operations were employed to extract local information, such as pig contours, textures, and postures, while the attention mechanism was used to model spatial correlations among different body regions. This architecture reduces the number of model parameters and computational complexity while enhancing feature representation under conditions involving densely distributed pigs, inclined postures, and inter-individual occlusion. It therefore provides stable target-location and behavioral features for subsequent oriented bounding box detection and pig multi-object tracking.

Let the input feature of the *l*-th EfficientViT block be *X_l_*. Its sandwich architecture can be expressed as

(1) $$X_{l+1}=\Phi_{F}^{\left(2\right)}\left[\Phi_{A}\left(\Phi_{F}^{\left(1\right)}\left(X_{l}\right)\right)\right]$$

where $\Phi_{F}^{\left(1\right)}$ and $\Phi_{F}^{\left(2\right)}$ denote the feed-forward networks before and after the self-attention layer, respectively, and Φ*_A_* denotes the cascaded group attention module.

EfficientViT employs a cascaded group attention mechanism that divides the input feature into *h* subfeatures along the channel dimension:

(2) *X* = [*X*_1_,*X*_2_,•••,*X_h_*]

The input to the *i*-th attention head is jointly constructed from the current subfeature and the output of the preceding attention head:

(3) $$X_{i}^{\prime }=\left\{\begin{array}{cc} X_{1}, & i=1\\ X_{i}+{\overset{⌢}{X}}_{i-1}, & \;2\leq i\leq h \end{array}\right\}$$

The corresponding attention operation is expressed as

(4) $${\overset{⌢}{X}}_{i}=\mathrm{Soft}\max\left(\frac{Q_{i}K_{i}^{T}}{\sqrt{d}}\right)V_{i}$$

where

(5) $$Q_{i}=X_{i}^{\prime }W_{i}^{Q},K_{i}=X_{i}^{\prime }W_{i}^{K},V_{i}=X_{i}^{\prime }W_{i}^{V}$$

Finally, the outputs of all attention heads are concatenated and linearly projected:

(6) $$Y=\mathrm{Concat}\left({\overset{⌢}{X}}_{1},{\overset{⌢}{X}}_{2},•••,{\overset{⌢}{X}}_{h}\right)W^{P}$$

where *Q_i_*, *K_i_*, and *V_i_* denote the query, key, and value matrices of the *i*-th attention head, respectively; $X_{i}^{\prime }$ denotes the input features of this head; $W_{i}^{Q}$, $W_{i}^{K}$, and $W_{i}^{V}$ denote the query, key, and value projection matrices, respectively; *d* is the feature dimension of a single attention head; and *W^P^* is the output projection matrix. Cascaded computation enables each subsequent attention head to use features extracted by the preceding head, reducing redundancy across attention heads and improving feature extraction efficiency.

#### 3.1.2. Dual-Teacher Knowledge Distillation Based on the Dinov3 Vision Foundation Model

**Figure 6:**
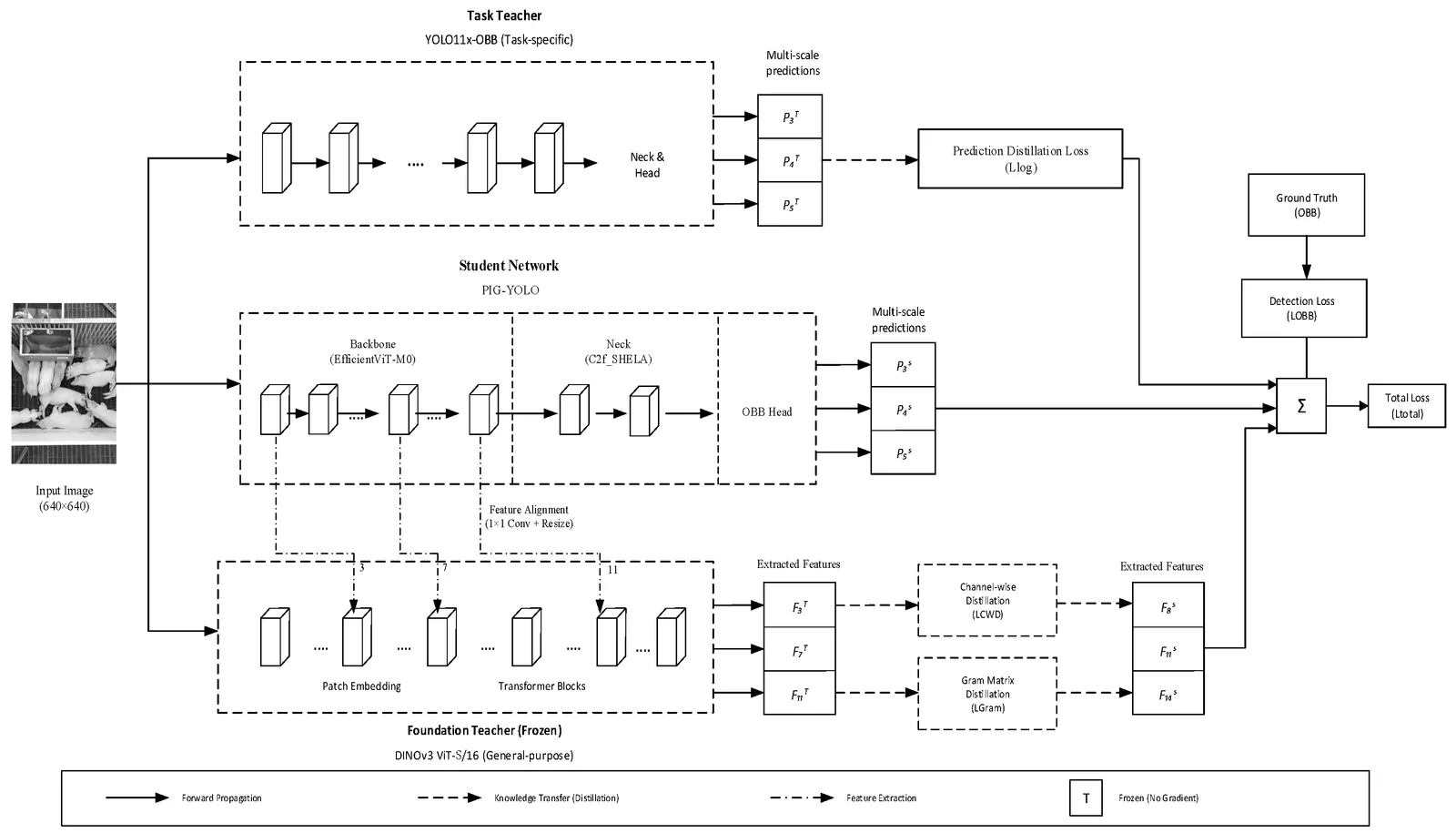
Dual-teacher knowledge distillation framework based on DINOv3.

To further improve the feature representation capability of the lightweight detection model under pig occlusion, inter-individual adhesion, and posture variations, a dual-teacher knowledge distillation method based on the DINOv3 vision foundation model was developed. YOLO11x-OBB was used as the task-specific teacher to transfer category-discrimination and oriented bounding box localization knowledge to the student model through its prediction responses. Meanwhile, a frozen DINOv3 ViT-S/16 model was introduced as the feature teacher. Its dense visual representations learned through large-scale pretraining were used to constrain the multi-scale feature learning of the student network. The proposed knowledge distillation framework is illustrated in Figure 6, and its training parameters are listed in Table 4.

For the task-teacher branch, let the prediction outputs of the teacher and student models be *P^T^* and *P^S^*, respectively. A response distillation loss based on the mean squared error is defined as

(7) $$L_{\log}=\frac{1}{N}{\left\|P^{S}-\mathrm{sg}\left(P^{T}\right)\right\|}_{2}^{2}$$

where *N* denotes the number of prediction elements and *sg*(⋅) denotes the stop-gradient operation. This loss encourages the student model to approximate the prediction distribution of the high-capacity teacher model, thereby improving the stability of pig behavior classification and oriented bounding box regression.

For the DINOv3 feature teacher, features were extracted from its 3rd, 7th, and 11th layers and were matched with the features from the 8th, 11th, and 14th layers of the student network, respectively. Considering the differences in channel dimensions and spatial resolutions between the two network architectures, feature mapping and resampling operations were first performed to align the heterogeneous features. Channel-wise knowledge distillation was then applied to constrain the spatial response distributions of the teacher and student features.

Let the normalized spatial response of the *c*-th channel in the *l*-th layer be

(8) $$P_{l,c}^{k}\left(i\right)=\frac{\exp\left(F_{l,c}^{k}\left(i\right)/\tau \right)}{\sum_{j=1}^{H_{l}W_{L}}\exp\left(F_{l,c}^{k}\left(j\right)/\tau \right)},\;k\in \left\{T,S\right\}$$

where *F* denotes the aligned features, and its superscript *k* in {*T*,*S*} indicates the network type. Here, *T* denotes the DINOv3 network serving as the feature teacher, and *S* denotes the PIG-YOLO student network. The subscript *l* indexes pairs of corresponding feature layers in the teacher and student networks, and *c* denotes the channel index. The value of *F* at spatial position *i* represents the feature response at that position. *H_l_* and *W_l_* denote the height and width of the aligned feature maps, respectively. Both *i* and *j* index spatial positions and range from 1 to the product of *H_l_* and *W_l_*, with *j* used as the summation index in the denominator.

The channel-wise distillation loss can then be expressed as

(9) $$L_{\mathrm{CWD}}=\frac{\tau^{2}}{L}\sum_{l=1}^{L}\frac{1}{C_{l}}\sum_{c=1}^{C_{l}}D_{\mathrm{KL}}\left(p_{l,c}^{T}\left\|p_{l,c}^{S}\right.\right)$$

where *τ* is the temperature coefficient, *C_l_* is the number of channels in the *l*-th layer, and *D_KL_*(⋅) denotes the Kullback–Leibler divergence.

To further transfer structural correlations among different feature channels, a Gram-matrix constraint was introduced. The feature map was flattened as ${\bar{F}}_{l}\in R^{C_{l}\times N_{l}}$, and its Gram matrix was defined as

(10) $$G\left(F_{l}\right)=\frac{1}{N_{l}}{\overset{-}{F}}_{l}{\overset{-}{F}}_{l}^{T},N_{l}=H_{l}W_{l}$$

The corresponding relational distillation loss was formulated as

(11) $$L_{\mathrm{Gram}}=\frac{1}{L}\sum_{l=1}^{L}\frac{1}{C_{l}^{2}}{\left\|G\left(F_{l}^{T}\right)-G\left(F_{l}^{S}\right)\right\|}_{F}^{2}$$

By integrating supervision from the ground-truth labels, response distillation from the task teacher, and feature distillation from the DINOv3 teacher, the overall optimization objective was defined as

(12) *L_total_* = *L_OBB_* + *λ*_log_*L*_log_ + *λ_DINO_*(*L_CWD_* + *βL_Gram_*)

where *L_OBB_* consists of the ProbIoU localization loss, classification loss, and distribution focal loss. According to the training configuration used in this study, *λ*_log_ = 0.5, *λ_DINO_* = 0.1 and *β* = 0.1. During training, the parameters of both teacher networks were frozen, and only the student model and feature-alignment modules were updated. During inference, only the student network was retained; therefore, the proposed distillation strategy introduced no additional computational overhead from the teacher models.

### 3.2. Improved AdaptiveSORT Model for Pig Multi-Object Tracking

The pig multi-object tracking algorithm follows the tracking-by-detection paradigm. The detection results produced by PIG-YOLO are used as inputs, while Kalman filtering and the Hungarian algorithm are employed to achieve continuous tracking of individual pigs. After the image at frame (T) is acquired, the detector first extracts the location, category, and confidence score of each pig. A camera motion compensation module is then applied to correct prediction errors caused by camera vibration or changes in viewpoint.

**Figure 7:**
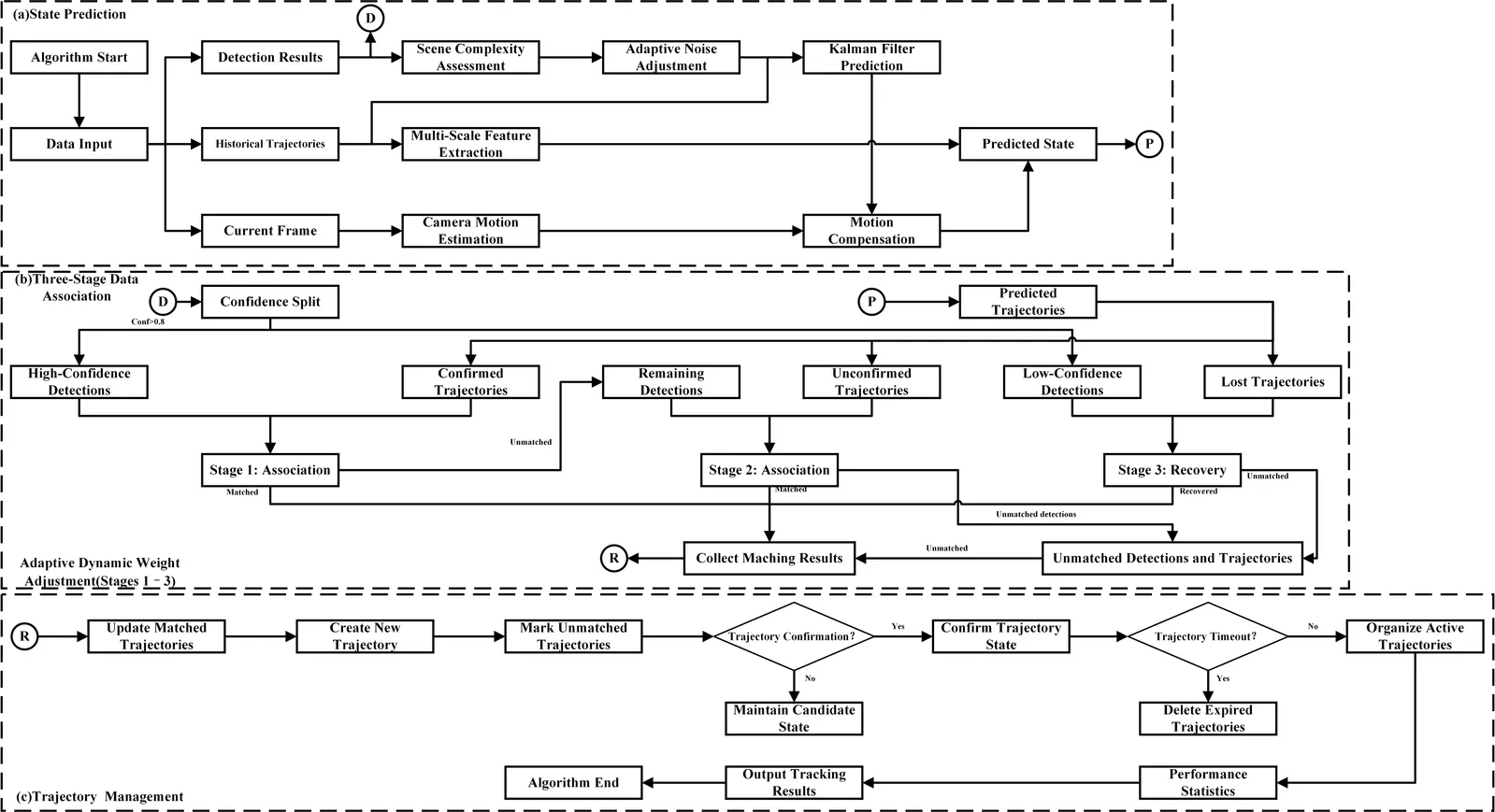
Overall workflow of the proposed AdaptiveSORT pig multi-object tracking algorithm.

On this basis, an adaptive prediction module is introduced to dynamically adjust the process noise of the Kalman filter according to scene complexity, thereby improving prediction stability in crowded scenes and under occlusion or rapid motion. Furthermore, to address identity loss caused by close contact, occlusion, and high appearance similarity among pigs, a multi-scale appearance feature extraction module is incorporated into the original SORT framework [26]. A three-stage data association strategy is also designed to progressively match high-confidence detections, unconfirmed tracks, and low-confidence detections. This strategy reduces the number of identity switches and improves tracking continuity. The overall workflow of the proposed algorithm is illustrated in Figure 7, and the operating principles of its core modules are described below.

#### 3.2.1. Camera Motion Compensation Module

The conventional SORT framework assumes a stationary camera, and camera translation or shake can therefore cause systematic drift in predicted positions. Although all videos in this study were recorded using fixed overhead cameras, minor vibrations of the camera mount or external disturbances may still cause slight global image shifts. Furthermore, the model is intended for future deployment on a rail-mounted inspection robot for mobile pig posture detection and continuous tracking. In this setting, apparent displacement induced by camera motion is superimposed on the pigs’ own motion, affecting trajectory prediction and identity association. To address this issue, AdaptiveSORT applies camera motion compensation between consecutive frames. Optical flow is used to estimate the homography matrix *H_t_* ∈ *R*^3×3^ between adjacent frames, and the Kalman filter prediction ${\hat{X}}_{t}={\left[x,y,w,h\right]}^{T}$ is projected into the motion-compensated coordinate system:

(13) $${\hat{X}}_{t}^{\prime }=H_{t}\cdot {\hat{X}}_{t}$$

This module effectively removes systematic errors caused by camera vibration or translation and corrects the predicted bounding box locations produced by the Kalman filter. Consequently, the predicted trajectories are more consistent with the actual motion of the targets.

#### 3.2.2. Adaptive Prediction Module

Even with camera motion compensation, a Kalman filter with fixed noise parameters may still perform poorly in complex scenes. Therefore, a scene-aware adaptive prediction module is designed to dynamically adjust the process noise of the Kalman filter according to scene complexity, thereby improving prediction accuracy.

First, a scene-complexity indicator is defined as

(14) *SC_t_* = *α* ⋅ *N*_det_ + *β* ⋅ *σ_motion_*

where *N*_det_ denotes the number of detection boxes in the current frame, *σ_motion_* denotes the velocity variance of the track set, and *α*, *β* is a weighting factor.

Second, based on the scene complexity indicator *SC_t_*, the process-noise covariance matrix of the Kalman filter is adaptively adjusted as follows:

(15) *Q_t_* = *Q*_0_ ⋅ (1 + *SC_t_* ⋅ *σ_velocity_*)

where *σ_velocity_* denotes the normalized track velocity and *Q*_0_ denotes the initial process-noise matrix.

In crowded scenes or scenes involving high-speed motion, the filter automatically increases the prediction uncertainty to accommodate abrupt changes in target motion. In relatively sparse scenes, a lower uncertainty is maintained to achieve more accurate state prediction.

#### 3.2.3. Multi-Scale Feature Extraction Module

Geometric and motion information alone is insufficient to resolve identity confusion caused by occlusion and highly similar appearances. Inspired by CNN backbone networks, this study introduces multi-scale feature learning and an attention mechanism to enhance target appearance representation. Spatial attention is specifically employed to emphasize discriminative spatial regions. Given an input feature map *F*, the weights generated by the spatial attention module are expressed as

(16) *A* = *σ*(*Conv*_1×1_(*F*))

The enhanced feature map is calculated as

(17) *F*^′^ = *A*⊙*F*

where ⊙ denotes element-wise multiplication.

Features {*f*^(^*^k^*^)^} are extracted at multiple scales, and the final feature representation is obtained through weighted fusion:

(18) $$f=\frac{\sum_{k}w_{k}f^{\left(k\right)}}{\sum_{k}w_{k}},\;w_{k}\propto \exp\left(-\tau \cdot u_{k}\right)$$

where *f*^(^*^k^*^)^ denotes the feature extracted at the *k*-th scale and *u_k_* denotes the uncertainty measure associated with that scale.

#### 3.2.4. Three-Stage Data Association Strategy

After target predictions and appearance features are obtained, AdaptiveSORT employs a three-stage progressive association mechanism. Hierarchical matching is performed to progressively confirm and recover tracks, thereby reducing identity switches and improving target recall. The association cost function is defined as

(19) $$C\left(i,j\right)=\alpha \left(SC_{t}\right)\cdot \left(1-\mathrm{IoU}\left(d_{t},t_{j}\right)\right)+\beta \left(SC_{t}\right)\cdot \|f\left(d_{i}\right)-f\left(t_{j}\right){\|}_{2}+\gamma \left(SC_{t}\right)\cdot {\tilde{D}}_{M}\left(d_{i},t_{j}\right)$$

where *d_i_* denotes a detection box, *t_j_* denotes a track, and ${\tilde{D}}_{M}$ denotes the normalized Mahalanobis distance. The association weights *α*, *β*, *γ* are dynamically adjusted according to scene complexity. In sparse scenes, greater emphasis is placed on geometric information, and the corresponding geometric weight *α* is therefore increased. In crowded scenes, appearance information *β* is assigned a greater weight to improve target discrimination.

The matching procedure of the three-stage data association strategy is described as follows.

**Stage 1:** High-confidence detections with confidence scores greater than 0.8 are matched with confirmed tracks.

**Stage 2:** The remaining detections are matched with unconfirmed tracks. At this stage, the intersection-over-union threshold is relaxed, and the contribution of appearance features is increased.

**Stage 3:** Low-confidence detections are matched with lost tracks to recover interrupted trajectories and reduce track fragmentation.

## 4. Model Training and Experimental Results

### 4.1. Training Environment

To ensure the consistency and reliability of model training, all experiments were conducted under the same hardware and software environment. The operating system was Windows 11, and the computer was equipped with an Intel(R) Core(TM) i5-14600KF CPU operating at 3.50 GHz and an NVIDIA GeForce GTX 5060 Ti GPU with 16 GB of memory. The experimental environment was developed using Python 3.9 and the PyTorch 2.9 deep learning framework, with CUDA 11.7 and cuDNN8.5.0 employed for computational acceleration.

The main training parameters were configured as follows. The input image size was set to 640 × 640 pixels. The initial and final learning rates were 0.01 and 0.001, respectively. Stochastic gradient descent (SGD) was selected as the optimizer, with a momentum of 0.937. The cosine annealing factor and weight decay coefficient were set to 0.1 and 0.0005, respectively. The batch size was 16, and the model was trained for 300 epochs. Mosaic data augmentation was disabled during the final 10 epochs.

### 4.2. Model Evaluation Metrics

#### 4.2.1. Evaluation Metrics for the Detection Model

Precision (*P*), recall (*R*), mean average precision (*mAP*), model size, frames per second (*FPS*), and floating-point operations (FLOPs) were used to comprehensively evaluate the performance of the detection model. The corresponding equations are given as follows:

(20) $$P=\frac{\mathrm{TP}}{\mathrm{TP}+\mathrm{FP}}\times 100\%$$

(21) $$R=\frac{\mathrm{TP}}{\mathrm{TP}+\mathrm{FN}}\times 100\%$$

(22) $$\mathrm{AP}=\int_{0}^{1}P\left(R\right)\mathrm{dr}$$

(23) $$\mathrm{mAP}=\frac{1}{M}\sum_{i=1}^{M}\mathrm{AP}\left(i\right)\times 100\%$$

(24) $$F1=2\times \frac{P\times R}{P+R}\times 100\%$$

(25) $$\mathrm{FPS}=\frac{N}{t}$$

where *TP*, *FP*, and *FN* denote the numbers of true positives, false positives, and false negatives, respectively; *AP* denotes the average precision for an individual category; *M* denotes the number of detection categories; and *N* denotes the number of images processed within a period of *t*. Precision and recall reflect the accuracy of model predictions and the ability of the model to detect target instances, respectively. The F1-score provides a comprehensive measure of the balance between precision and recall, whereas *mAP* evaluates the overall detection accuracy of the model. Model size reflects the storage and deployment requirements of the model, while *FPS* is used to evaluate its real-time inference capability. Considering that the model is intended for deployment on an NVIDIA Jetson Nano edge device, FLOPs were further employed to evaluate computational complexity, where 1 GFLOPs = 10^9^ FLOPs. Provided that the requirements for detection accuracy and real-time performance are satisfied, a smaller model size and lower computational cost are more favorable for deployment on resource-constrained platforms.

#### 4.2.2. Evaluation Metrics for the Multi-Object Tracking Algorithm

Multi-object tracking accuracy (*MOTA*), multi-object tracking precision (*MOTP*), the number of identity switches (*IDs*), identity F1-score (*IDF*1), and higher-order tracking accuracy (*HOTA*) were used to evaluate the tracking performance.

(26) $$\mathrm{MOTA}=1-\frac{\sum \left(\mathrm{FN}+\mathrm{FP}+\mathrm{IDs}\right)}{\sum \mathrm{GT}}\epsilon \left(-\infty ,1\right]$$

(27) $$\mathrm{MOTP}=\frac{\sum_{i,t}d_{i,t}}{c_{t}}$$

(28) $$\mathrm{IDF}1=\frac{2\mathrm{IDTP}}{2\mathrm{IDTP}+\mathrm{IDFP}+\mathrm{IDFN}}$$

(29) $${\mathrm{HOTA}}_{\alpha }=\sqrt{\frac{\sum_{c\in TP_{\alpha }}A_{\alpha }\left(c\right)}{\left|TP_{\alpha }\right|+\left|FN_{\alpha }\right|+\left|FP_{\alpha }\right|}}=\sqrt{{\mathrm{DetA}}_{\alpha }\cdot {\mathrm{AssA}}_{\alpha }}$$

where *FN_t_*, *FP_t_*, *IDs_t_*, and *GT_t_* denote the numbers of false negatives, false positives, identity switches, and ground-truth targets in the *t*-th frame, respectively. *c_t_* denotes the number of correctly matched predicted and ground-truth targets in the *t*-th frame, and *d_i_*_,_*_t_* denotes the localization error between the *i*-th pair of matched targets. *IDTP*, *IDFP*, and *IDFN* denote the numbers of identity true positives, identity false positives, and identity false negatives, respectively. *DetA* and *AssA* denote detection accuracy and association accuracy, respectively.

*MOTA* provides an overall assessment of false negatives, false positives, and identity switches. *MOTP* evaluates target localization accuracy, whereas *IDs* measures the frequency of identity switches during tracking. *IDF*1 reflects the ability of the tracker to preserve target identities, and *HOTA* jointly evaluates detection and association performance. Higher *MOTA*, *IDF*1, and *HOTA* values indicate better tracking performance, whereas a lower number of identity switches is preferable. When *MOTP* is defined in terms of localization distance error, a lower *MOTP* value indicates more accurate target localization.

### 4.3. Results and Analysis of Pig Multi-Object Detection

#### 4.3.1. Ablation Experiments

To evaluate the effectiveness of the proposed pig detection model, ablation experiments were conducted on the test dataset, and the results are presented in Table 5. As shown in the table, after replacing the original backbone of the baseline YOLO11m-OBB model with the lightweight EfficientViT backbone, precision and recall decreased by 2.2 and 0.4 percentage points, respectively, whereas mAP50 increased by 0.3 percentage points. Meanwhile, the number of parameters, FLOPs, and model size were reduced by approximately 34.9%, 50.5%, and 32.9%, respectively. These results demonstrate that EfficientViT can substantially reduce model complexity while largely preserving detection performance. After the DINOv3-based dual-teacher knowledge distillation strategy was further introduced, all accuracy-related metrics improved markedly. Compared with the baseline model, precision, recall, mAP50, and mAP50–95 increased by 3.7, 9.9, 8.0, and 8.9 percentage points, respectively. Overall, the proposed improvements not only significantly enhance the detection performance of the model but also effectively reduce its parameter count and computational cost.

#### 4.3.2. Comparative Experiments

To investigate the effects of different detection models, lightweight backbone networks, and DINO teacher models on pig detection performance, a series of comparative experiments were conducted. The results are presented in Table 6, Table 7 and Table 8. As shown in Table 6, the proposed model achieved the best overall performance among all the compared detection models. Compared with the baseline model, the proposed model improved precision, recall, mAP50, and mAP50–95 by 3.7, 9.9, 8.0, and 8.9 percentage points, respectively. Meanwhile, the number of parameters, FLOPs, and model size were reduced by approximately 37.7%, 51.5%, and 36.2%, respectively. These results demonstrate that the proposed model can substantially improve detection accuracy while reducing computational cost. As shown in Table 7, among the evaluated lightweight backbone networks, EfficientViT-M0 achieved the highest mAP50 and mAP50–95 values, reaching 85.4% and 74.7%, respectively. Compared with the baseline model, its parameter count, FLOPs, and model size were reduced by approximately 34.9%, 50.5%, and 32.9%, respectively, indicating a favorable balance between detection accuracy and model lightweightness. As shown in Table 8, the distillation performance varied considerably among the different DINO teacher models, with DINOv3 ViT-S/16 achieving the best performance. Compared with the EfficientViT-based model without knowledge distillation, the model distilled using DINOv3 ViT-S/16 improved precision, recall, mAP50, and mAP50–95 by 5.9, 10.3, 7.7, and 10.9 percentage points, respectively.

Overall, EfficientViT-M0 effectively reduced model complexity, while knowledge distillation using DINOv3 ViT-S/16 further enhanced the feature representation and detection capabilities of the model. Consequently, the proposed model achieved a favorable balance between detection accuracy and computational efficiency.

### 4.4. Results and Analysis of Pig Multi-Object Tracking

#### 4.4.1. Comparative Experiments of Tracking Models

To comprehensively evaluate the performance of the improved tracking algorithm, a series of comparative experiments were conducted on both the public and private datasets by combining different detection models with multiple tracking algorithms. The results are presented in Table 9 and Table 10. On the public dataset, PIG-YOLO+AdaptiveSORT achieved HOTA and MOTA scores of 96.32% and 98.82%, respectively, with no identity switches. Compared with PIG-YOLO+BoT-SORT, PIG-YOLO+ByteTrack, and PIG-YOLO+DeepSORT, the proposed combination improved HOTA by 0.83, 19.52, and 6.32 percentage points, respectively, and MOTA by 1.78, 22.72, and 6.95 percentage points, respectively. Meanwhile, the number of identity switches was reduced by 2, 2, and 4, respectively. On the private dataset, PIG-YOLO+AdaptiveSORT achieved a HOTA score of 94.98% and a MOTA score of 97.31%, with only one identity switch. Compared with PIG-YOLO+BoT-SORT, PIG-YOLO+ByteTrack, and PIG-YOLO+DeepSORT, HOTA increased by 1.25, 20.24, and 6.69 percentage points, respectively, while MOTA increased by 1.69, 23.69, and 7.59 percentage points, respectively. The number of identity switches was reduced by 3, 4, and 5, respectively. These experimental results demonstrate that the combination of PIG-YOLO and AdaptiveSORT achieves superior tracking accuracy and identity preservation on both the public and private datasets, with better overall tracking performance than the other evaluated detector–tracker combinations.

#### 4.4.2. Generalization Experiment of the Tracking Model

To evaluate the generalization capability of the proposed tracking model more comprehensively, the publicly available piglet detection and tracking dataset released by Wutke et al. [27] was used as an external test set. Collected at the Futterkamp experimental farm in Germany, this dataset records the daily activities of group-housed piglets in pens with slatted floors. It includes daytime and nighttime imaging conditions and scenes involving feeding, resting, and interactions among individuals. The number of piglets and the extent of mutual occlusion vary across videos. The videos were recorded from an overhead view using a fixed AXIS M3024-LVE camera mounted approximately 3 m above the floor, with an original resolution of 1280 × 800 pixels and a frame rate of 10 frames/s. The PIG-YOLO detector was kept unchanged. In addition to the original baseline methods, including ByteTrack, BoT-SORT, and DeepSORT, representative trackers such as OC-SORT, StrongSORT, and BoostTrack were included to more comprehensively assess the adaptability and identity association capabilities of different methods in external settings. Retaining the original baselines while broadening the comparison further tested whether AdaptiveSORT maintained its performance advantage when the data source changed. The results are presented in Table 11.

As shown in Table 10, PIG-YOLO+AdaptiveSORT achieved HOTA, MOTA, and IDF1 scores of 71.231%, 76.043%, and 83.008%, respectively, with only 17 identity switches, outperforming the other methods overall. Compared with ByteTrack, one of the stronger baselines, it increased HOTA by 0.054 percentage points and reduced identity switches by 11. Compared with BoT-SORT, it improved HOTA, MOTA, and IDF1 by 0.559, 0.202, and 1.513 percentage points, respectively, and reduced identity switches by 3. AdaptiveSORT also achieved the best association accuracy (AssA), MOTP, precision, and recall scores, reaching 71.934%, 73.848%, 90.701%, and 85.951%, respectively. These results indicate that PIG-YOLO+AdaptiveSORT maintains good detection, track association, and identity preservation performance in cross-dataset evaluation, demonstrating the generalization capability of the proposed model.

#### 4.4.3. Visualization and Comparison of Tracking Results

**Figure 8:**
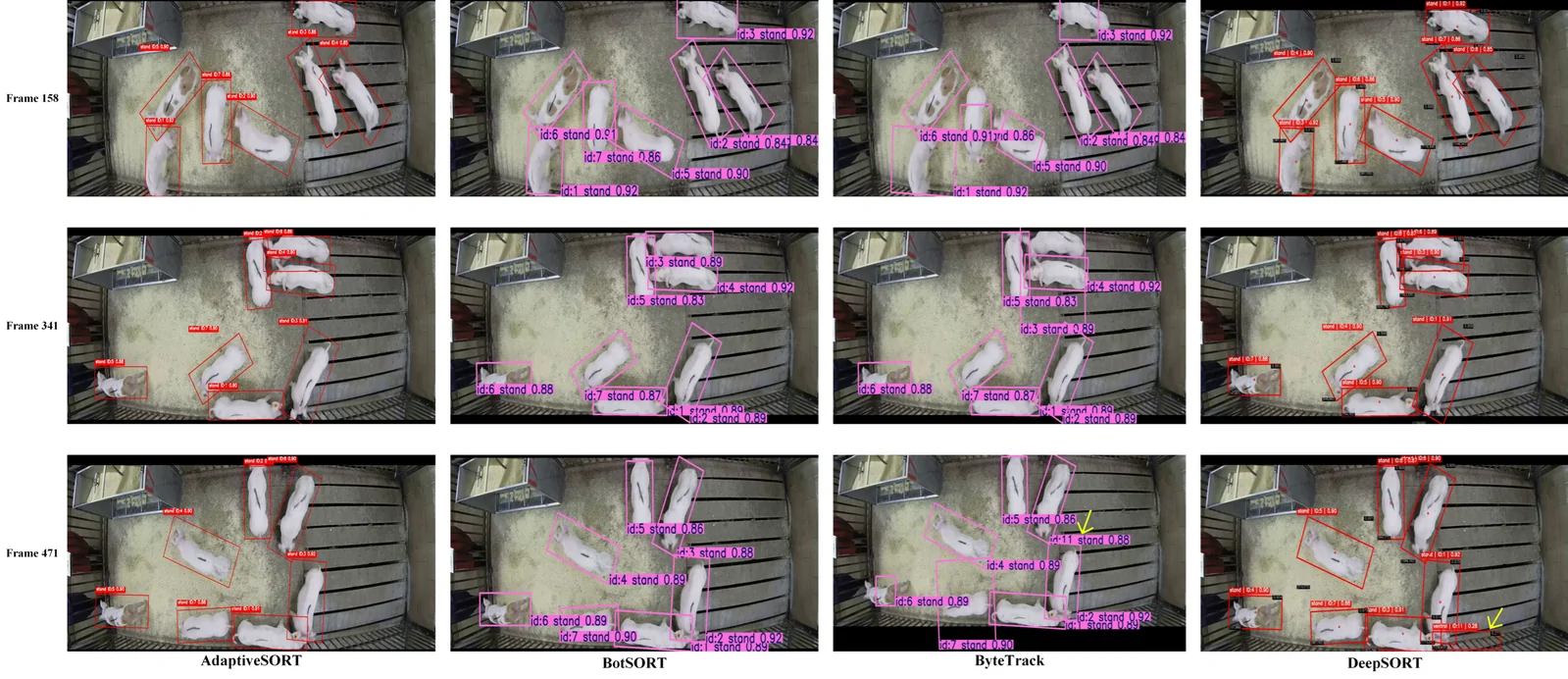
Comparison of tracking results between PIG-YOLO+AdaptiveSORT and other methods on the public dataset.

**Figure 9:**
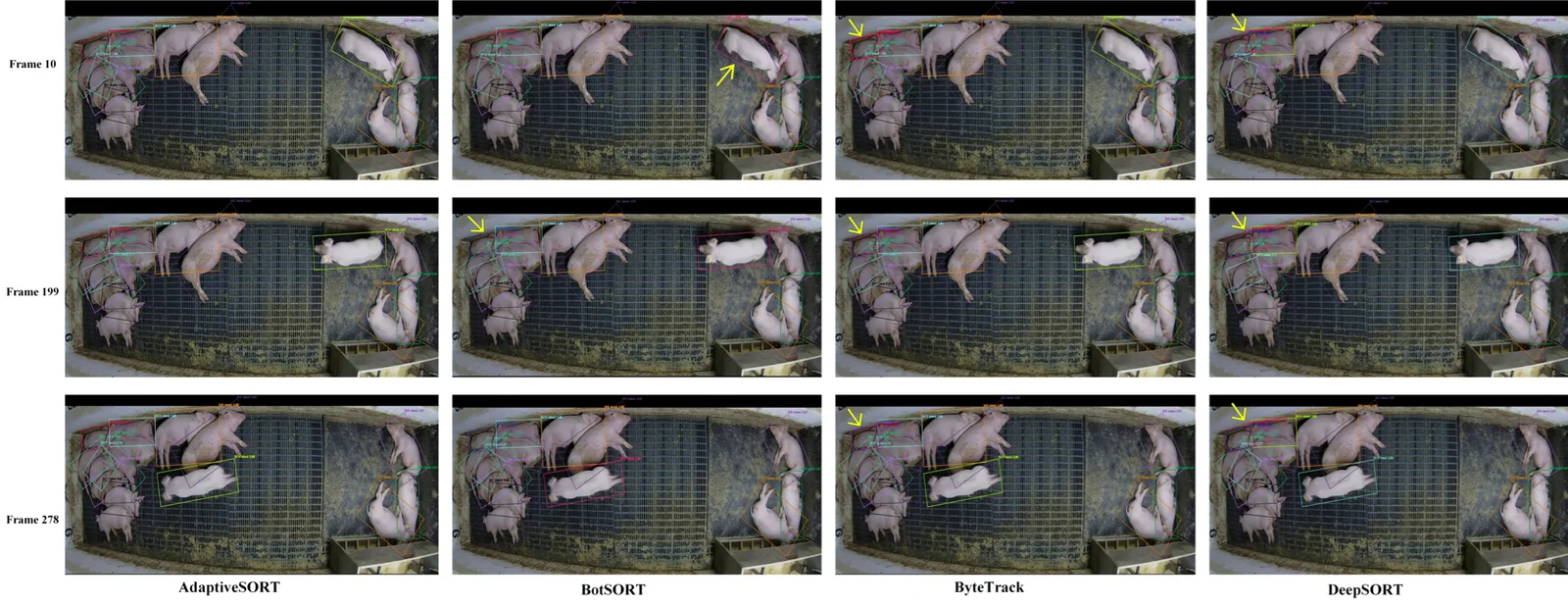
Comparison of tracking results between PIG-YOLO+AdaptiveSORT and other methods on the private dataset.

To further provide an intuitive comparison of the advantages and improvements achieved by the proposed algorithm in pig tracking, Frames 158, 341, and 471 from the public dataset and Frames 10, 199, and 278 from the private dataset were selected for visualization. The tracking results are shown in Figure 8 and Figure 9.

On the public dataset, all algorithms successfully tracked the pigs in Frames 158 and 341. However, identity assignment errors occurred for ByteTrack and DeepSORT in Frame 471. Specifically, ByteTrack generated an anomalous target ID, while DeepSORT exhibited inconsistent identity association. In contrast, AdaptiveSORT maintained continuous target identities across the different frames, and its oriented detection boxes showed good agreement with the body orientations of the pigs.

In the private dataset, the pigs were mainly concentrated on the left side of the pen, resulting in severe occlusion and close contact among adjacent individuals. AdaptiveSORT maintained stable pig identities in Frames 10, 199, and 278 without obvious identity assignment errors. In comparison, BoT-SORT produced incorrect identity associations in Frames 10 and 199. ByteTrack and DeepSORT also exhibited varying degrees of ID mismatching in the densely populated region on the left side of the pen, as indicated by the arrows in Figure 9, suggesting weaker trajectory association stability under occlusion.

Overall, compared with the other algorithms, the proposed AdaptiveSORT algorithm exhibits superior tracking performance and stronger adaptability to complex environments, enabling stable tracking of individual pigs.

### 4.5. Pig Behavior Statistics

For test video A003, the pig behavior statistics generated by PIG-YOLO+AdaptiveSORT are presented in Table 12. The results include the temporal distributions of eight behavioral categories for 16 individual pigs. As shown in table, standing was the predominant behavior in this video, accounting for 34.17% of the total behavioral duration. Standing while feeding and lateral lying were the next most frequent behaviors, accounting for 15.52% and 14.48%, respectively. Ventral lying, lying, sitting, and standing while drinking accounted for 10.52%, 9.27%, 6.46%, and 5.31%, respectively. Because A003 represents a relatively sparse scene with a high activity level, standing, feeding, and other active behaviors accounted for relatively large proportions of the total observation time. These results demonstrate that the proposed method can quantify the duration of different behaviors for individual pigs during continuous tracking, thereby providing a data basis for analyzing activity patterns and monitoring the health status of pigs.

The behavioral durations recorded for individual pigs were further aggregated by video to compare group behavioral composition across scenes. The time proportion of each behavior was calculated as its cumulative duration divided by the total duration of all behaviors recorded across all individuals in the corresponding video. Summaries for the public and private datasets are presented in Table 13 and Table 14, respectively.

As shown in Table 13, standing accounted for the largest proportion in both A001 and A003, at 36.11% and 34.17%, respectively, while feeding while standing accounted for 17.56% and 15.52%, respectively. In contrast, lateral lying, ventral lying, and lying accounted for relatively high proportions in A002, A004, and A005. In A005, these three lying categories together accounted for 81.21% of the total recorded behavioral duration. These clips exhibited different distributions of behavioral durations, encompassing both active scenes with greater time allocated to standing and feeding and resting scenes dominated by lying postures.

Table 14 shows similar differences in behavioral composition. In B003, standing and feeding while standing accounted for 37.02% and 17.98%, respectively, totaling 55.00%. In B001, B002, B004, and B005, lateral lying accounted for the largest proportion, at 28.33%, 31.33%, 32.27%, and 27.38%, respectively. Together, the individual-level duration records in Table 12 and the video-level summaries in Table 13 and Table 14 enable the characterization of both behavioral differences among individuals and group behavioral composition across clips, providing a basis for subsequent analysis of pig activity patterns and health monitoring.

## 5. Discussion

The results of this study demonstrate that the combination of the PIG-YOLO detector and AdaptiveSORT tracker provides robust performance for the detection and multi-object tracking of group-housed pigs.

First, compared with YOLO11m-OBB and the other detection models, PIG-YOLO improved pig detection performance while further reducing model complexity. Although the lightweight EfficientViT backbone reduces redundant computation during feature extraction, replacing the backbone alone may introduce fluctuations in the representation of certain fine-grained features. Therefore, a dual-teacher knowledge distillation strategy was employed to jointly optimize the lightweight student model. The task-specific teacher transfers knowledge related to pig category discrimination and oriented bounding box localization, whereas the DINOv3 feature teacher enhances the feature representation of the student model through multi-layer dense semantic supervision. This strategy compensates for part of the information loss caused by the lightweight backbone and improves the model’s adaptability to variations in pig posture. Because the two teacher models are used only during training and only the student model is retained during inference, no additional computational overhead is introduced during actual detection.

Second, comparisons with BoT-SORT, ByteTrack, and DeepSORT demonstrated that AdaptiveSORT provides better overall tracking performance. Based on the SORT framework, AdaptiveSORT incorporates camera motion compensation, adaptive prediction, multi-scale appearance features, and three-stage data association. Camera motion compensation corrects prediction errors caused by image vibration or changes in viewpoint, while the adaptive prediction module dynamically adjusts the process noise of the Kalman filter according to scene complexity, resulting in more stable estimation of target motion states. Multi-scale appearance features improve the discrimination of pigs with highly similar appearances, whereas the three-stage data association strategy progressively matches high-confidence detections, unconfirmed tracks, and low-confidence detections. These improvements optimize the association cost between detections and tracks and consequently improve the results of Hungarian matching, thereby reducing identity switches and trajectory interruptions under occlusion and high-density conditions and enhancing identity preservation in group-housing environments. The PIG-YOLO+AdaptiveSORT tracking framework can not only accomplish pig detection and identity tracking in complex group-housing environments but can also be integrated with behavior-statistics algorithms to establish a continuous analytical pipeline from target localization and individual association to behavioral-duration aggregation. For example, stable trajectories can be used to quantify behaviors such as standing, feeding, drinking, and lying for individual pigs. Such information can provide objective data for analyzing individual activity patterns, adjusting feeding management strategies, and assessing health status, while reducing the subjective errors associated with manual observation.

Nevertheless, the present study examines relatively simple behavioral patterns in group-housed pigs, with limited consideration of interactions among individuals and the effects of external conditions. Future research will further investigate associations between tracking failures and pig breed, age in days, body mass, stocking density, and housing conditions to inform algorithm optimization across farming environments. Behavioral analysis could be extended to include interactive behaviors such as aggression, chasing, and play. Continuous trajectories could also be used to examine approaches and separations between individuals and changes in activity rhythms, enabling investigation of social relationships among group-housed pigs. Comparing behavioral durations and distribution patterns between pigs with and without abnormalities could further support health assessment and early warning of abnormal behavior. Meanwhile, the lightweight design was evaluated primarily in terms of parameter count, computational cost, and model size. Future work could investigate model quantization, inference acceleration, and real-time performance on edge devices such as the NVIDIA Jetson Nano to balance deployment costs and monitoring effectiveness. Furthermore, all data in this study were collected using fixed overhead cameras, which help reduce occlusion among pigs and perspective distortion. However, the model’s adaptability to oblique viewpoints and cross-camera settings requires further validation. Future research could incorporate monitoring from multiple viewpoints and investigate tracking methods that fuse information across cameras to improve the system’s applicability and scalability in large-scale pig farms.

## 6. Conclusions

In this study, a multi-object tracking method for group-housed pigs was developed by integrating the PIG-YOLO detector with the AdaptiveSORT tracker. In the detection experiments, PIG-YOLO achieved precision, recall, mAP50, and mAP50–95 values of 94.7%, 88.3%, 93.1%, and 85.6%, respectively, representing improvements of 3.7, 9.9, 8.0, and 8.9 percentage points over the baseline model. Meanwhile, the number of parameters, GFLOPs, and model size were reduced by 37.7%, 51.5%, and 36.2%, respectively. On the public test dataset, PIG-YOLO+AdaptiveSORT achieved HOTA, MOTA, and IDF1 scores of 96.32%, 98.82%, and 99.41%, respectively, with no identity switches. On the private dataset, the corresponding scores were 94.98%, 97.31%, and 98.86%, respectively, with only one identity switch. Compared with combinations of PIG-YOLO with BoT-SORT, ByteTrack, and DeepSORT, the proposed method demonstrated superior overall performance in terms of detection accuracy, trajectory association, and identity preservation. In conclusion, PIG-YOLO+AdaptiveSORT not only improves pig multi-object detection and tracking performance but also achieves model lightweighting. Its robust and stable tracking capability is essential for accurately quantifying the duration and distribution of specific behaviors, thereby providing highly reliable and actionable statistical information for real-time health monitoring and management of group-housed pigs.

## Figures and Tables

**Table 1:** Characteristics of the test datasets.

Datasets	Video ID	Daytime	Nighttime	Sparse	Dense	Duration/min	Activity Level	Numbers
Public Datasets	A001	✓	✗	✗	✓	1	High	15
	A002	✗	✓	✓	✗		Low	10
	A003	✓	✗	✓	✗		High	16
	A004	✓	✗	✗	✓		Low	6
	A005	✗	✓	✗	✓		Low	11
Private Datasets	B001	✓	✗	✗	✓	1	Low	6
	B002	✗	✓	✓	✗		Low	10
	B003	✓	✗	✓	✗		High	28
	B004	✗	✓	✗	✓		Low	11
	B005	✓	✗	✗	✓		Low	14

**Table 2:** Classification criteria and descriptions of pig behaviors.

Behavior Category	Classification Criteria	Label
Lateral lying	The pig lies on either its left or right side with all four legs clearly visible on one side of the body and the abdomen partially exposed.	lateral
Feeding	The head is lowered toward the feed trough. The forelegs may be either flexed or extended, and the body is positioned to allow the mouth to reach the feed.	eat
Lying	The forelegs are extended forward on the ground, while the hind legs are visible on either the left or right side. The abdomen is in contact with the ground and the back faces upward.	lie
Ventral lying	Both the forelegs and hind legs are folded beneath the body, with the abdomen in contact with the ground and the back facing upward. The limbs are completely obscured from view.	ventral
Drinking	The head is lowered toward the drinker, with the neck extended. The forelegs may be slightly flexed, and the body is oriented toward the water source.	drink
Standing	The body is supported by all four vertically extended legs, with the back facing upward and the abdomen downward. All four hooves are in contact with the ground.	stand
Sitting	The trunk is approximately upright relative to the extended forelegs, with the hindquarters in contact with the ground.	sit
Foreleg kneeling	The forelegs are flexed and kneeling on the ground, with the chest in contact with or close to the ground, while the hind legs remain in a standing position.	fkp

**Table 3:** Dataset split and distribution of behavior classes in the annotated images.

Label	Training Instances	Training Images	Validation Instances	Validation Images	Test Instances	Test Images	Total Instances
lateral	8131	1409	1016	176	1016	176	10,163
eat	2993	1229	374	153	374	153	3741
lie	2702	1157	337	145	337	145	3376
ventral	4451	1417	556	177	556	177	5563
drink	212	178	27	22	27	22	266
stand	6624	1439	827	180	827	180	8278
sit	877	593	109	74	109	74	1095
fkp	76	72	9	9	9	9	94

**Table 4:** Training parameters of the dual-teacher knowledge distillation framework.

Training Parameter	Setting
Student model	PIG-YOLO
Task teacher model	YOLO11x-OBB
Foundation teacher model	DINOv3 ViT-S/16
Pretraining dataset	LVD-1689M
Foundation teacher layers	3, 7, 11
Student distillation layers	8, 11, 14
Image size	640 × 640
Number of training epochs	300
Batch size	16
Optimizer	SGD
Response distillation loss	L2
Feature distillation loss	CWD + Gram
Distillation weight schedule	Constant
Localization loss	ProbIoU
Inference stage	Student model only

**Table 5:** Results of the ablation experiments.

Baseline	EfficientViT	DINOv3	P/%	R/%	mAP50/%	mAP50–95/%	Params/M	FLOPs/G	ModelSize/MB
✓			91.0	78.4	85.1	76.7	20.91	71.9	42.3
✓	✓		88.8	78.0	85.4	74.7	13.61	35.6	28.4
✓		✓	90.6	81.9	90.8	80.5	20.88	71.4	42.4
✓	✓	✓	94.7	88.3	93.1	85.6	13.03	34.9	27.0

**Table 6:** Comparative results of different detection models.

Models	P/%	R/%	mAP50/%	mAP50–95/%	Model Size/MB	GFLOPs	Parameters/M
YOLO3-tiny-obb	85.8	78.8	84.3	71.3	20.3	15.2	10.05
YOLO5m-obb	84.5	66.6	75.3	63.6	51.8	66.2	25.69
YOLO6m-obb	82.5	72.7	77.8	68.2	103.3	158.9	51.43
YOLO8m-obb	79.1	67.1	76.4	62.2	53.2	80.9	26.42
YOLO9m-obb	88.7	78.4	86.4	77.2	35.7	63.5	17.55
YOLO10m-obb	90.4	77.7	84.9	75.8	32.3	61.0	15.91
Baseline	91.0	78.4	85.1	76.7	42.3	71.9	20.91
YOLO12m-obb	79.3	75.5	79.6	68.7	42.5	70.8	20.99
YOLO26m-obb	83.4	72.3	79.6	69.1	47.5	71.6	23.48
RTDETR-x-obb	91.8	78.2	86.3	74.8	127.5	228.8	63.34
RF-DETR-large-obb	88.3	82.3	84.7	73.6	128.5	100.6	33.64
DEIM-DFINE-obb	93.5	65.5	84.8	71.1	40.7	24.9	3.19
DEIM-DFINE-StarNet-obb	90.2	72.5	83.7	69.2	40.0	22.9	3.24
DEIM-DFINE-MobileNetV3-obb	91.8	77.5	85.1	71.1	35.5	20.7	2.81
PIG-YOLO	94.7	88.3	93.1	85.6	27.0	34.9	13.03

**Table 7:** Comparative results of different lightweight backbone networks.

Models	P/%	R/%	mAP50/%	mAP50–95/%	Model Size/MB	GFLOPs	Parameters/M
Baseline	91.2	78.4	85.1	76.7	42.3	71.9	20.91
+FasterNet-T0	85.2	79.8	83.9	73.3	28.3	37.0	13.85
+MobileNetV3-Small	89.2	75.5	83.6	73.6	26.4	32.3	12.87
+MobileNetV4-Small	83.3	76.3	82.1	70.4	32.4	49.1	15.84
+RepViT-M0.9	85.7	78.0	82.3	71.5	33.8	45.4	16.46
+StarNet-S50	89.2	75.8	83.1	72.5	24.1	32.5	11.75
+ConvNeXtV2-Atto	86.8	75.0	81.6	71.3	31.1	40.4	15.34
+EfficientViT-M0	88.8	78.0	85.4	74.7	28.4	35.6	13.61

**Table 8:** Distillation results using different DINO teacher models.

Baseline+EfficientViT	P/%	R/%	mAP50/%	mAP50–95/%	Model Size/MB	GFLOPs	Parameters/M
+dinov2_vitl14	91.4	79.1	85.6	76.5	27.0	34.9	13.03
+dinov2_vitb14	87.7	81.3	86.2	77.0			
+dinov2_vits14	90.7	79.9	85.7	76.6			
+dinov3_vitl16	89.6	79.6	86.0	76.7			
+dinov3_vits16plus	92.0	81.8	87.2	81.7			
+dinov3_vitb16	92.4	87.2	92.2	83.2			
+dinov3_vits16	94.7	88.3	93.1	85.6			

**Table 9:** Performance comparison of different detector–tracker combinations on the public dataset.

Models	Trackers	HOTA	DETA	ASSA	MOTA/%	MOTP/%	IDF1/%	IDs	P/%	R/%
YOLOv8m-OBB	BOTSORT	94.07	96.51	91.69	96.40	26.88	88.23	3	98.71	97.75
	ByteTrack	76.43	78.42	74.50	75.48	28.82	87.25	8	87.40	88.42
	DeepSORT	92.12	94.51	89.78	94.36	26.74	97.18	0	97.29	97.06
	AdaptiveSORT	94.15	96.60	91.77	96.52	27.63	98.27	0	97.68	98.86
YOLOv9m-OBB	BOTSORT	96.04	98.53	93.61	98.48	27.46	97.83	2	99.74	98.79
	ByteTrack	78.80	80.85	76.81	78.77	28.80	88.44	1	89.30	89.53
	DeepSORT	91.55	93.93	89.23	93.75	27.05	96.87	0	96.98	96.75
	AdaptiveSORT	96.16	98.66	93.73	98.65	27.92	99.33	0	99.20	99.46
YOLOv12m-OBB	BOTSORT	92.99	95.41	90.64	95.21	28.18	83.66	7	99.31	96.04
	ByteTrack	78.29	80.33	76.31	77.83	29.36	88.34	7	88.33	89.86
	DeepSORT	91.00	93.36	88.69	92.96	27.90	95.01	5	95.87	97.28
	AdaptiveSORT	94.74	97.20	92.34	97.13	29.03	98.58	0	97.72	99.46
YOLOv26m-OBB	BOTSORT	75.68	77.64	73.76	77.09	27.71	61.02	21	99.70	77.83
	ByteTrack	72.70	74.59	70.86	71.90	28.34	78.50	7	89.22	81.97
	DeepSORT	88.69	90.99	86.44	90.41	27.24	83.81	13	96.80	93.82
	AdaptiveSORT	88.40	90.70	86.17	90.57	27.17	91.59	3	99.30	91.28
DEIM	BOTSORT	90.27	92.62	87.99	92.25	29.76	82.7	10	98.17	94.24
	ByteTrack	77.63	79.65	75.66	77.33	31.19	88.67	0	88.62	88.72
	DeepSORT	87.77	90.05	85.55	89.43	29.02	93.53	4	94.74	94.79
	AdaptiveSORT	91.89	94.27	89.56	94.10	30.64	97.05	0	96.99	97.11
**PIG-YOLO**	BOTSORT	95.49	97.97	93.07	97.04	27.53	97.12	2	97.87	99.24
	ByteTrack	76.80	78.79	74.85	76.10	28.38	81.02	2	87.65	88.63
	DeepSORT	90.00	92.34	87.72	91.87	26.89	91.77	4	95.22	96.83
	**AdaptiveSORT**	**96.32**	**98.82**	**93.88**	**98.82**	**27.23**	**99.41**	**0**	**99.57**	**99.53**

Bold indicates the best-performing combination.

**Table 10:** Performance comparison of different detector–tracker combinations on the private dataset.

Models	Algorithms	HOTA	DetA	AssA	MOTA/%	MOTP/%	IDF1/%	IDs	P/%	R/%
YOLOv8m-OBB	BOTSORT	92.11	94.88	89.42	94.72	27.36	95.42	5	97.46	96.18
	ByteTrack	74.65	76.55	72.80	72.84	29.31	84.62	11	85.31	86.95
	DeepSORT	90.06	92.74	87.46	92.31	27.18	95.36	2	95.82	95.11
	AdaptiveSORT	92.60	95.21	90.06	95.04	28.05	97.36	1	96.34	97.88
YOLOv9m-OBB	BOTSORT	94.08	96.82	91.42	96.75	27.89	96.18	4	98.58	97.65
	ByteTrack	76.73	78.62	74.88	76.46	29.25	86.21	4	87.26	87.92
	DeepSORT	89.93	92.06	87.84	91.62	27.41	95.21	2	95.74	95.18
	AdaptiveSORT	94.62	97.2	92.11	97.12	28.26	98.52	1	98.14	98.63
YOLOv12m-OBB	BOTSORT	90.94	93.62	88.33	92.71	28.61	81.92	10	97.84	94.92
	ByteTrack	75.91	78.25	73.64	75.16	29.81	86.54	10	86.12	88.03
	DeepSORT	88.76	91.21	86.37	90.64	28.30	93.28	7	94.22	95.86
	AdaptiveSORT	92.83	95.64	90.11	95.82	29.42	97.31	1	96.08	98.61
YOLOv26m-OBB	BOTSORT	72.76	75.32	70.28	73.94	28.23	57.84	26	98.61	74.1
	ByteTrack	70.18	72.41	68.02	68.83	28.92	75.42	10	87.36	79.21
	DeepSORT	85.53	88.02	83.11	87.12	27.76	81.02	16	95.12	91.47
	AdaptiveSORT	85.34	87.84	82.92	87.35	27.68	89.46	5	98.42	89.73
DEIM	BOTSORT	87.66	90.25	85.14	89.43	30.21	80.54	13	96.92	92.87
	ByteTrack	74.93	77.10	72.83	74.58	31.74	86.12	3	86.73	87.26
	DeepSORT	85.55	88.23	82.95	86.91	29.48	91.75	6	93.08	93.62
	AdaptiveSORT	89.61	92.08	87.21	91.78	31.06	95.64	1	95.42	96.37
**PIG-YOLO**	BOTSORT	93.73	96.35	91.18	95.62	27.94	95.88	4	96.74	98.06
	ByteTrack	74.74	77.02	72.53	73.62	28.86	78.92	5	85.10	86.57
	DeepSORT	88.29	91.03	85.64	89.72	27.31	89.64	6	93.84	95.12
	**AdaptiveSORT**	**94.98**	**97.48**	**92.55**	**97.31**	**27.66**	**98.86**	**1**	**98.74**	**98.61**

Bold indicates the best-performing combination.

**Table 11:** Results of the tracking model generalization experiment.

Trackers	HOTA	DetA	AssA	MOTA/%	MOTP/%	IDF1/%	IDs	P/%	R/%
ByteTrack	71.177	70.633	71.774	69.904	73.798	81.111	28	84.447	85.748
BoostTrack	68.575	70.096	67.169	72.994	73.771	82.795	21	87.529	85.168
BOTSORT	70.672	70.019	71.403	75.841	73.662	81.495	20	90.439	84.849
DeepOCSORT	70.522	69.991	71.106	75.185	73.829	80.265	21	89.354	85.398
DeepSort	69.990	69.174	70.866	61.156	73.824	58.308	1497	83.322	79.931
HybridSort	70.494	69.650	71.395	67.993	73.797	79.588	23	82.752	84.789
OCSORT	70.167	69.631	70.754	62.66	73.817	57.473	1679	81.978	84.309
SFSORT	70.167	69.925	70.474	75.951	73.828	81.696	21	90.680	84.671
StrongSORT	70.180	69.666	70.747	75.659	73.611	80.128	23	90.625	84.437
**AdaptiveSORT**	**71.231**	**70.607**	**71.934**	**76.043**	**73.848**	**83.008**	**17**	**90.701**	**85.951**

Bold indicates the best-performing combination.

**Table 12:** Temporal distribution of eight behavioral categories for 16 pigs in video A003.

Behavior	The Statistics Table of Pig Behavior Information/Unit: s															
	ID0	ID1	ID2	ID3	ID4	ID5	ID6	ID7	ID8	ID9	ID10	ID11	ID12	ID13	ID14	ID15
lateral	20	6	5	14	4	10	10	7	5	6	10	2	15	10	5	10
eat	11	7	17	18	2	4	5	18	13	7	9	7	5	12	8	6
lie	3	3	5	2	7	9	4	7	4	6	5	7	5	14	5	3
ventral	1	12	3	9	1	13	9	3	9	5	4	10	5	7	5	5
drink	1	2	4	1	4	2	2	3	5	1	2	10	7	0	3	4
stand	21	23	21	9	36	20	21	20	12	26	20	20	21	12	23	23
sit	2	2	4	6	5	1	2	0	6	4	7	2	1	4	9	7
fkp	1	5	1	1	1	1	7	2	6	5	3	2	1	1	2	2

**Table 13:** Percentage of time spent on each pig behavior in the public dataset.

Behavior Category	A001	A002	A003	A004	A005
stand	36.11	10.67	34.17	12.50	7.73
eat	17.56	5.17	15.52	4.44	5.15
drink	6.33	3.00	5.31	4.44	1.06
lateral	11.11	34.83	14.48	28.06	38.48
ventral	10.33	23.5	10.52	22.22	24.70
lie	8.44	15.67	9.27	18.06	18.03
sit	5.33	3.83	6.46	5.28	3.03
fkp	4.78	3.33	4.27	5.00	1.82

**Table 14:** Percentage of time spent on each pig behavior in the private dataset.

Behavior Category	B001	B002	B003	B004	B005
stand	13.89	12	37.02	8.64	14.17
eat	9.72	3.33	17.98	5.76	9.52
drink	1.94	2	4.94	2.58	2.86
lateral	28.33	31.33	13.87	32.27	27.38
ventral	20.56	24.5	9.82	23.94	22.98
lie	18.61	20.17	6.67	18.48	14.17
sit	3.89	4.33	6.25	4.7	6.79
fkp	3.06	2.33	3.45	3.64	2.14

## Data Availability

The training code and data associated with this article can be found in the project repository at https://github.com/GodLu0510/A-Multi-Object-Tracking-Method-for-Pigs-Based-on-Oriented-Bounding-Box-Detection.git (accessed on 28 September 2026).
